# Neuroimaging, Behavioral, and Gait Correlates of Fall Profile in Older Adults

**DOI:** 10.3389/fnagi.2021.630049

**Published:** 2021-02-18

**Authors:** Krystal M. Kirby, Sreekrishna Pillai, Robert M. Brouillette, Jeffrey N. Keller, Alyssa N. De Vito, John P. Bernstein, Arend W. A. Van Gemmert, Owen T. Carmichael

**Affiliations:** ^1^Fine Motor Control and Learning Laboratory (FMCL), School of Kinesiology, Louisiana State University, Baton Rouge, LA, United States; ^2^Biomedical Imaging Center, Pennington Biomedical Research Center, Baton Rouge, LA, United States; ^3^Institute for Dementia Research and Prevention, Pennington Biomedical Research Center, Baton Rouge, LA, United States; ^4^Department of Psychology, Louisiana State University, Baton Rouge, LA, United States

**Keywords:** falls, gait, cognitive, fMRI, motor function

## Abstract

Prior research has suggested that measurements of brain functioning and performance on *dual tasks* (tasks which require simultaneous performance) are promising candidate predictors of fall risk among older adults. However, no prior study has investigated whether brain function measurements during dual task performance could improve prediction of fall risks and whether the type of subtasks used in the dual task paradigm affects the strength of the association between fall characteristics and dual task performance. In this study, 31 cognitively normal, community-dwelling older adults provided a self-reported fall profile (number of falls and fear of falling), completed a gait dual task (spell a word backward while walking on a GaitRite mat), and completed a supine dual task (rhythmic finger tapping with one hand while completing the AX continuous performance task (AX-CPT) with the other hand) during functional magnetic resonance imaging (fMRI). Gait performance, AX-CPT reaction time and accuracy, finger tapping cadence, and brain functioning in finger-tapping-related and AX-CPT-related brain regions all showed declines in the dual task condition compared to the single task condition. Dual-task gait, AX-CPT and finger tapping performance, and brain functioning were all independent predictors of fall profile. No particular measurement domain stood out as being the most strongly associated measure with fall variables. Fall characteristics are determined by multiple factors; brain functioning, motor task, and cognitive task performance in challenging dual-task conditions all contribute to the risk of falling.

## Introduction

Falls affect more than 30% of older adults. Therefore, it is not surprising that falls in this population are the leading cause of non-fatal injury in this population (Centers for Disease Control and Prevention, 2014), while also a major cause of fatal injury. Falls are also associated with declines in functional status and social activity (Stel et al., 2004), as well as significant financial burdens due to consequent health care utilization (Alexander et al., 1992; Hoffman et al., 2017). Unfortunately, identifying older adults at increased risk of falling is especially challenging. A large set of studies have assessed fall risk using a variety of different predictors. Studies have assessed fall risk based on gait and slip responses (Rahul and Thurmon, 2017), gait and fall history (Toulotte et al., 2006; Ansai et al., 2017), musculoskeletal function tests (MacRae et al., 1992), and cognitive performance (Hausdorff et al., 2001; Verghese et al., 2002; Springer et al., 2006; Holtzer et al., 2007; Herman et al., 2010; Buracchio et al., 2011). Despite some studies showing some promise as to its usefulness to predict falls using these measurements, it still has not been proven to be flawless; hence the vast number of studies using different metrics to assess fall risk. Most studies are able to discriminate with modest accuracy fallers from non-fallers. For example, one posturography-based approach discriminated multiple-fallers from non-multiple-fallers with 85% accuracy (Howcroft et al., 2017). Improving accuracy of fall risk prediction systems is especially important, because interventions targeted to high-fall-risk individuals have the potential to reduce fall risk through strength and balance training, built environment modification, and other methods [for a complete list, see Stevens and Burns (2015)].

One key limitation of measurements that focus on a single domain, such as cognition, motor function, or sensation, is that they do not simulate realistic scenarios in which complex motor tasks must be completed at the same time as a distracting and unrelated cognitive task. Distraction scenarios are common among older adults–consider walking while answering a cell phone–and frequently lead to falls. Laboratory-based dual task paradigms combining gait with a cognitive load have been devised to simulate such scenarios (Hausdorff et al., 2001; Springer et al., 2006; Allali et al., 2007; Yogev-Seligmann et al., 2008; Herman et al., 2010; MacAulay et al., 2014, 2015; Ansai et al., 2017; Rahul and Thurmon, 2017). Other dual-task paradigms have focused on concurrent execution of two, unrelated motor tasks (Toulotte et al., 2006). While prior studies have identified associations between these dual-task measures and falls, it is unclear how these measures compare to competing measures, such as brain functional measures, in terms of strength of association with falls.

Functional neuroimaging during performance of dual tasks provides an alternative means of assessing fall risk by identifying deficiencies in the functioning of brain networks that contribute to dual task performance, even in the absence of overt deficiencies in task performance. Brain function deficiency measures are promising as fall predictors because these inadequacies could culminate in cognitive and motor deficiencies in more varied and complex real-world environments occurring outside the laboratory. Furthermore, when brain function deficiencies are identified, often, without intervention, they become progressively worse over time. Although previous research has assessed the brain functional correlates of dual-task performance, including the rhythmic motor components of distracted gait (Holtzer et al., 2011; Van Impe et al., 2011; Johnson and Shinohara, 2012; Doi et al., 2013; Leone et al., 2017; Papegaaij et al., 2017), and brain activity during walking and reciting letters (Verghese et al., 2017) to our knowledge none of these studies determined whether the brain functional measures predicted fall risk based on retrospective fall history.

The goal of this study was to take a step toward improved fall prediction by assessing performance on a gait dual task, brain functioning during a stationary dual task, and performance on the stationary dual task as an independent correlate with fall history. We collected the stationary task data from 31 cognitively normal older adults after they had already completed a minimum of three yearly clinical evaluations, each of which included a self-reported fall profile and gait during dual task performance. To our knowledge, this is the first study that simultaneously assessed distracted gait measures as well as performance and brain functional measures on a stationary dual task, to identify correlates with fall history.

## Materials and Methods

### Participants

All participants were enrolled in the Louisiana Aging Brain Study (LABrainS), a longitudinal observational cohort maintained by the Institute for Dementia Research and Prevention (IDRP) at the Pennington Biomedical Research Center (PBRC) designed to investigate cognitive, motor, and affective changes among cognitively normal older adults aged 60–85. Participants throughout Louisiana were recruited into LABrainS through typical media sources such as newspaper advertisements and television, in addition to outreach done by the IDRP. Exclusion criteria from LABrainS included: a history of neurological or untreated health conditions (e.g., Parkinson’s disease and/or a traumatic brain injury) that might cause cognitive impairment, or a Geriatric Depression Scale ≥ 6 [15 item version, (Yesavage et al., 1982]. A total of 416 LaBrains participants had at least 3 years of complete gait, neuropsychological, and fall data at the time this study started. A set of 50 of these individuals were recruited into the current study. Additional exclusion criteria for this study were contraindications to MRI, left handedness, and vision not corrected to 20/20. Of the 50 participants, six did not provide analyzable MRI data due to excessive head motion or similar acquisition issues. An additional 13 participants did not follow task directions and therefore did not provide analyzable data. Therefore, data was processed and analyzed from the remaining 31 participants; see Supplementary Figure 1. Informed consent was obtained from participants prior to their visit assessments. This study was approved by the PBRC Institutional Review Board. Characteristics of the 31 participants are shown in Table 1. Average age for the participants was 73.0 ± 6.7 years, with 25 females and six males. Cognitive and motor status was assessed using the Repeatable Battery for the Assessment of Neuropsychological Status (RBANS) (Randolph et al., 1998) and the Short Physical Performance Battery (SPPB) (Guralnik et al., 1994), respectively.

**TABLE 1:** LABrainS participants characteristics and demographics.

Age	73.0 ± 6.7 years
Sex	25 female, 6 male
Ethnicity	30 white, 1 black/African American
RBANS	111.9 ± 17.6
SPPB	11.14 ± 1.60
Falls yes/no	14 yes, 17 no
Number of falls ever	1.48 ± 2.56
Fear of falling	0.19 ± 0.37
Time between successive gait measurements	12.39 ± 1.86 months
Time between most recent gait measurement and fMRI acquisition	2.19 ± 4.27 months

### Fall History

Fall history among LaBrainS participants was collected at each yearly clinical visit. Since the term “fall” may have different meanings to different individuals, the definition used in this study was consistent with established recommendations: “times in which an individual unexpectedly lost balance and unintentionally came to rest upon the ground, floor, or other object” (Lamb et al., 2005). This did not include those times that participants were able to regain their balance before coming into contact with the ground. Each participant completed a survey of fall history at each yearly visit. Collected data items identified fear of falling (as a binary), times fallen in the past 12 months, times fallen in the past 2 years, total times fallen over the lifespan, and categorization of the person as a “faller” or a “non-faller” (i.e., a person who did or did not report at least one fall over the lifespan). This retrospective fall data was included in analysis.

### Gait Dual Task Acquisition and Analysis

Gait data was acquired at each clinical visit. The GAITRite system (CIR Systems, Inc., Sparta, NJ, United States), an electric sensor walkway, was used for collection of gait data. In the single task condition, participants were instructed to walk across the walkway “using their normal everyday walking speed.” In the dual task condition, participants were additionally instructed to spell a word backward aloud while walking (for list of words, see the GAITRite manual). Stride length (the line of progression between two consecutive footprints of the same foot) and step time (time elapsed between the contact of one foot on the floor to the opposite foot’s contact) served as gait performance measures in single and dual task conditions. Average stride length and step time within the single and dual task conditions were calculated at each visit. In our analysis, we used these specific gait variables: average stride length for single task, dual task, and dual-task hit, average step time for single task, dual task, and dual-task hit. GAITRite data has been analyzed successfully in older adult cohorts (MacAulay et al., 2014; Brown et al., 2015).

### Stationary Dual Task Design

Participants performed two tasks with button boxes, separately and then simultaneously, while lying supine in an MRI machine. Button boxes were held in the left and right hands over the chest area with the middle and index fingers of each hand being used to perform the tasks. The first task was self-paced finger tapping for 90 s. On the instruction screens, participants were told to tap in cadence with a flashing box that appeared for 0.03 s every 0.4 s, alternating the tapping between left middle and index fingers, for a total of 10 s, before being told to tap at a natural cadence without a visual cue. The second task was the AX-continuous performance task (AX-CPT), a streaming letter memorization and recognition task (Servan-Schreiber et al., 1996). In each trial of this task, participants viewed a pair of letters serially on the screen; a white letter (“cue”) followed by a blue letter (“probe”), both appearing on a black background. The instruction was to press the right index finger button when the probe letter was X immediately following an A cue. The left index finger button should be pressed for any other combination of cue and probe letters. There were four types of trials, depending on the cue and probe letter: AX trials (A cue, X probe), BX trials (non-A cue, X probe), AY trials (A cue, non-X probe), and BY trials (non-A cue, non-X probe). Non-A cues were drawn from this set of letters: E, P, G, R, S, and V. Non-X probes were drawn from this set of letters: F, J, M, Q, and U. The cue was shown for 0.5 s, then disappeared to a black screen for a 5.5 s response period, then the probe was shown for 0.5 s, followed by a 5.5 s response period. Participants completed 36 AX-CPT trials in the single task condition. Next, the finger tapping and AX-CPT tasks were performed simultaneously, consisting of 36 AX-CPT trials with the right hand and self-paced finger tapping with the left hand. Behavioral data was acquired with MATLAB 2016a with Psychophysics Toolbox and the Fiber Optic Response Pad (FORP) button box system on non-auto release button mode. A timestamp was generated every 10 ms indicating if a button was currently depressed, and this data was analyzed using in-house software. Only the first button press was recorded as a response.

### Stationary Dual Task Behavior Analysis

For the finger tapping task, the average number of taps per second (tapping cadence), its standard deviation, and the time the button was held down were calculated. For the AX-CPT task, we calculated the overall accuracy as the number of cues and probes answered correctly divided by the total number of cues and probes. This was further categorized into accuracy by trial type. Reaction time was the time difference between the onset of the cue or probe and when the button was pressed. The dual-task hit was the percentage change going from single task to dual task, and this was calculated for accuracy and reaction time for each trial type, as well as overall. For finger tapping, we calculated tap cadence as the mean number of seconds in between taps. We calculated tap cadence within a sliding window of 15 s duration, and calculated tap consistency as the standard deviation of tap cadence across all sliding window locations. We also calculated tap duration as the mean amount of time (in seconds) that the button was held down during a tap. Each of these measures was calculated separately in single and dual task conditions. The dual-task hit was also calculated for each of the tapping measures.

### fMRI Acquisition

Functional magnetic resonance imaging (fMRI) scans were acquired on a 3T MRI scanner (General Electric, 750 W Discovery, 32-channel quadrature head coil) using a blood oxygen level dependent echo-planar imaging (BOLD-EPI) pulse sequence. Participants wore both a pulse oxygenation sensor and respiratory monitoring belt during scanning to correct for cardiac and respiratory influences on fMRI signals. Key acquisition parameters were the following: voxel size: 3.4 mm × 3.4 mm × 3.5 mm, TR: 3 s, number of slices: 52, and TE: 30 ms. Structural images required for functional data analysis were obtained using a T1-weighted magnetization-prepared gradient echo pulse sequence with the following parameters: TR: 8.7 ms, TE: 3.8 ms, FA: eight degrees, number of slices: 176, and voxel size: 1 mm × 1 mm × 1 mm.

### fMRI Data Analysis

Functional MRI data was analyzed with MATLAB R2016a and the Statistical Parametric Mapping 12 (SPM12) toolbox. Preprocessing steps included these steps: realignment for head motion correction, co-registration to the structural scan, slice timing corrections, smoothing using a 6 mm full width at half maximum Gaussian kernel, and warping to the Montreal Neurological Institute (MNI) template. We used the RETROICOR algorithm to remove cardiac and respiratory components of each time series. Time points representing volumes with excessive head motion (defined as greater than 1.5 mm of translation or 1.5 degrees of rotation from the previous time point) and activation spike artifacts (defined as global mean brain activation greater than 2.3 standard deviations above the mean across all time points) were removed from analysis. The data for each participant was entered into a first-level voxel-wise analysis using the general linear model. Each trial was modeled as a boxcar function convolved with the canonical hemodynamic response function that began at the onset of the stimulus presentation. First level beta maps, performed at the single participant level, quantified differences in BOLD signal between different components of the single and dual task. Region-of-interest (ROI) analysis was performed using ROIs obtained from other studies (Witt et al., 2008; Lesh et al., 2013; Lopez-Garcia et al., 2016), see Supplementary Table 1. For each ROI, the set of contrast values within a 5 mm radius sphere surrounding the ROI location was averaged to provide the ROI summary. These ROI-level summary measures were the primary fMRI measures of interest in the statistical analysis.

### Statistical Analysis: Analysis of Dual-Task Hits

For each of the behavioral, fMRI, and gait summary variables, we calculated the mean and standard deviation across participants in single-task and dual-task conditions. We quantified the “dual-task hit” for each measure in terms of the signed percent difference in the measure between single-task and dual-task conditions. We used one-sample *T* tests to assess whether the means of such dual-tasks hits differed significantly from zero. Among behavioral measures, we then assessed correlations between corresponding reaction time and accuracy dual task hits to explore whether participants tended to exhibit decrements in speed, accuracy, or both. In addition, we assessed correlations between AX-CPT dual task hits and finger tap dual task hits to explore whether participants tended to experience performance decrements in one of the tasks or both simultaneously.

### Statistical Analysis: Predictors of Fall Profile

We took an incremental model building approach to assessing gait, behavioral, and imaging predictors of fall profile (Carmichael et al., 2012). Specifically, for each of the predictor variables, separate linear regression models were estimated with that variable as the sole predictor and one of the fall variables (presence or absence of fall history, number of falls over the past year, fear of falling, and total number of falls) as the outcome. Independent predictor variables entered into the single predictor models included the single-task, dual-task, and dual-task hit of accuracy, reaction time, tap cadence, tap duration, and tap consistency. *P* values less than 0.05 in those models were viewed as statistically significant. For fall outcomes with statistically significant regression models from more than one measurement domain (gait, behavior, imaging), a combined model was estimated including the most significant (i.e., lowest *P* value) predictor among the significant predictors in that domain. The combined models sought to assess whether any specific measurement domain showed a pattern of relatively stronger association with the fall variables, compared to other domains. Power was determined by using a correlation power analysis.

## Results

### Stationary Dual Task Performance

**FIGURE 1:**
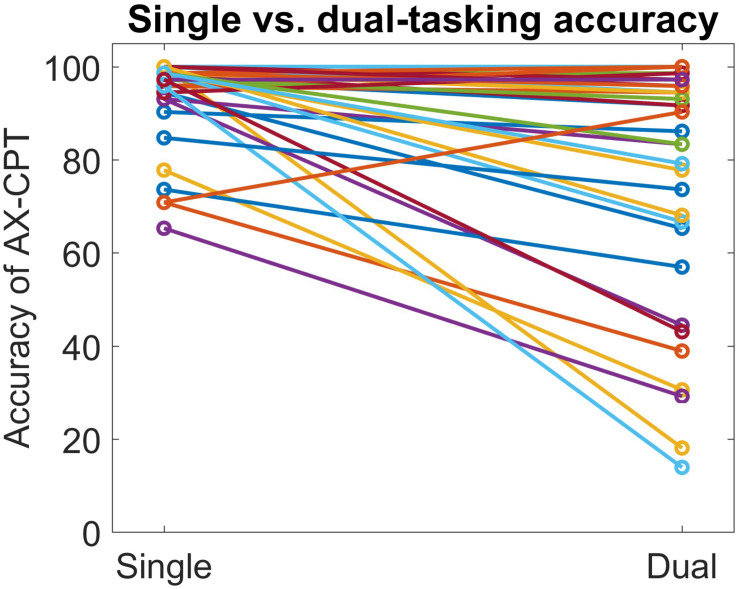
Scatterplot depicting inter-individual variability in dual task hits to AX-CPT accuracy.

AX-continuous performance task accuracy decreased significantly in the dual task condition, compared to the single task condition, both overall and in every trial type except BY (see Table 2 and Supplementary Table 2). In addition, AX-CPT reaction time increased significantly in the dual-task condition compared to the single-task condition (see Table 2), but this effect was mainly driven by increased reaction time on the BY trials. Tap cadence was slower, tap consistency was greater, and tap duration was longer in the dual-task condition compared to the single-task condition. Although the AX-CPT accuracy dual-task hit was substantial on average, there was marked inter-individual variability, with some participants maintaining high levels of accuracy (Figure 1). There was limited evidence of a significant relationship between dual-task hits to AX-CPT performance and dual-task hits to finger tap performance (see Supplementary Table 3).

**TABLE 2:** AX-CPT accuracy, AX-CPT reaction time, and finger-tapping performance in the single and dual task conditions, along with their standard errors.

	Single	St. error	Dual	St. error	% diff	*p*-value
AX-CPT accuracy	94.6	1.4	82.6	3.6	–12.7	< 0.001**
AX-CPT reaction time (s)	0.74	0.04	0.80	0.04	9.0	0.04*
Tap cadence (s/tap)	0.48	0.02	0.54	0.03	12.6	0.001**
Tap consistency (s/tap)	0.05	0.01	0.25	0.04	429.1	< 0.001**
Tap duration (s)	0.23	0.02	0.29	0.02	25.4	0.001**

Percent difference in the mean value and *p*-values for one-sample *T* tests are shown in the right column. * = significant at the *p* = 0.05 level, ** = significant at the *p* = 0.001 level.

### Stationary Dual Task fMRI Data

**FIGURE 2:**
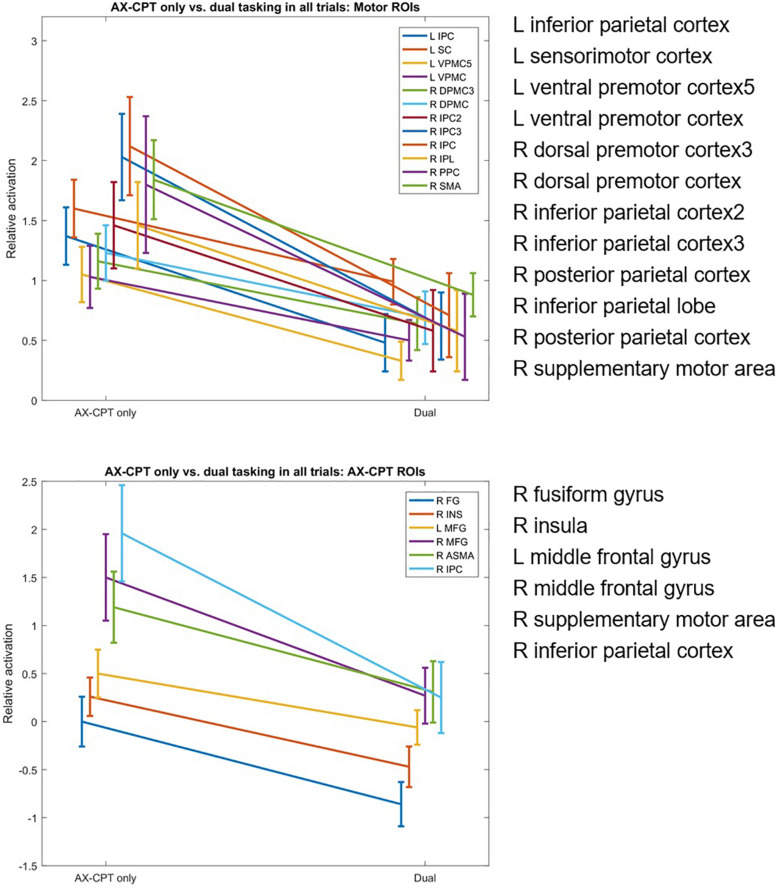
Dual-task hits in fMRI activation in AX-CPT-related and finger-tap-related regions of interest, for single AX-CPT task vs. dual AX-CPT/tapping task.

Eighteen of the pre-defined ROIs showed significantly reduced fMRI activation in the dual-task condition compared to the AX-CPT single-task condition. Of these ROIs, six were AX-CPT ROIs and 12 were finger tapping ROIs (Figure 2). No ROIs showed significantly greater fMRI activation in the dual-task condition compared to the AX-CPT single-task condition. When the fMRI data was partitioned into AX trials vs. non-AX trials, a similar pattern of reduced ROI activation emerged within each partition separately (see Supplementary Figure 2).

### Gait Dual Task Data

Gait step length decreased in the dual task (walking plus cognitive task) compared to the single task by 7.3% (*p* < 0.001). Gait step time increased significantly by 6.2% (*p* < 0.001). In addition, the standard deviations of both the step length and step time increased during the dual task compared to the single task, by 4.5 and 8.0%, respectively.

### Individual Predictors of Fall Profile

Multiple predictors, spanning multiple measurement domains, were significantly associated with each of the four fall profile variables in single-predictor regression models (see Table 3). Step lengths in the single- and dual-task conditions were significant predictors from the gait domain, while dual-task hits to finger tap cadence and an AX-CPT reaction time variable were significant predictors from the behavioral domain. 12 imaging ROIs (four AX-CPT ROIs and eight motor cortex ROIs) had dual-task fMRI hits that were significant predictors of total number of falls, while two imaging ROIs (one AX-CPT and one motor cortex ROI) had dual task hits that were significant predictors of faller/non-faller status. Specifically, the right superior parietal cortex ROI (of the AX-CPT set) had a dual task hit that correlated with three of our fall variables. No gait dual-task hit variables were correlated with falls.

**TABLE 3:** Correlations between each fall metric, shown in the upper left of each sub table, and each significant gait, behavioral, and imaging measure.

	Gait			Behavioral			Imaging			All		
Fear of falling	Single predictor model			Single predictor model			Single predictor model			Multiple predictor model		
	*p*	β	95% CI	*p*	β	95% CI	*p*	β	95% CI	*p*	β	95% CI
Tap cadence DTH	–	–	–	0.02	−0.04	−0.08, −0.01	–	–	–	0.004	−0.04	−0.08, −0.02
L SMA	–	–	–	–	–	–	0.02	−0.16	−0.29, −0.03	0.004	−0.18	−0.30, −0.06
Adjusted *R*^2^	–			0.14			0.18			0.39		
*F* (for Δ*R*^2^)	–			5.71			6.21			8.45		

**Falls in past year**	**Single predictor model**			**Single predictor model**			**Single predictor model**			**Multiple predictor model**		
	***p***	**β**	**95% CI**	***p***	**β**	**95% CI**	***p***	**β**	**95% CI**	***p***	**β**	**95% CI**

Gait dual step length	0.02	−0.06	−0.10, −0.02	–	–	–	–	–	–	0.12	−0.04	−0.09, 0.01
Tap cadence DTH	–	–	–	0.03	−0.03	−0.06, −0.003	–	–	–	0.02	−0.04	−0.07, −0.01
R sup parietal cortex	–	–	–	–	–	–	0.02	−0.05	−0.10, −0.01	0.05	−0.04	−0.08, 0.001

Adjusted *R*^2^	0.13			0.11			0.14			0.29		
*F* (for Δ*R*^2^)	5.42			4.78			5.84			4.88		
**Total number of falls**	**Single predictor model**			**Single predictor model**			**Single predictor model**			**Multiple predictor model**		
	***p***	**β**	**95% CI**	***p***	**β**	**95% CI**	***p***	**β**	**95% CI**	***p***	**β**	**95% CI**

Gait dual step length	0.02	−0.10	−0.18, −0.02	–	–	–	–	–	–	0.03	−0.08	−0.16, −0.01
R inf parietal cortex	–	–	–	–	–	–	0.02	−0.11	−0.20, −0.02	0.04	−0.09	−0.17, −0.01

Adjusted *R*^2^	0.15			–			0.15			0.25		
*F* (for Δ*R*^2^)	6.11			–			6.01			5.90		
**Faller/non-faller (binary)**	**Single predictor model**			**Single predictor model**			**Single predictor model**			**Multiple predictor model**		
	***p***	**β**	**95% CI**	***p***	**β**	**95% CI**	***p***	**β**	**95% CI**	***p***	**β**	**95% CI**

BY trials reaction time DTH	–	–	–	0.04	−0.008	−0.01, −0.001	–	–	–	0.02	−0.01	−0.01, −0.001
R DPMC3	–	–	–	–	–	–	0.04	−0.03	−0.05, −0.001	0.03	−0.03	−0.05, −0.004
Adjusted *R*^2^	–			0.10			0.10			0.22		
*F* (for Δ*R*^2^)	–			4.33			4.53			5.17		

*Measures listed have a significant correlation (*p* < 0.05) with the fall measure in a single predictor model and are therefore entered into the multiple predictor model shown in the far right column. *P*-values (p), regression coefficients (β), and 95% confidence intervals (CI) are shown for each model, in addition to the adjusted *R*^2^ and *F* values. Empty sub tables represent a fall measure and a measurement domain that did not yield any significant predictors for each single predictor model. DTH, dual-task hit; L SMA, left supplementary motor area; R DPMC, right dorsal premotor cortex.*

### Simultaneous Predictors of Fall Profile Variables

In multiple predictor models containing the most-significant gait, behavioral, and imaging predictors, there was no clear pattern suggesting that predictors from one domain or another provided the strongest associations with fall profile variables. In the multiple-predictor model with fear of falling as the outcome measure, both a stationary dual-task performance measure (tap cadence dual task hit) and an fMRI variable (L SMA dual task hit) were significant predictors with nearly equivalent *p* values (*p* = 0.004 for each), which shows increased power from the single-predictor models. In the multiple-predictor model with falls in the past year as the outcome measure, only one stationary dual-task performance measure (tap cadence dual task hit) was a significant predictor (*p* = 0.02). In the multiple-predictor model with total number of falls as the outcome measure, both of the gait dual task or fMRI predictor variables were significant predictors. In the multiple-predictor model with faller/non-faller status as the outcome variable, only one stationary dual task performance variable (BY reaction time dual task hit) was a significant predictor from the imaging domain, and the right dorsal premotor cortex three ROI was significant from the imaging domain. The power of these multiple predictor models was over 80% in all cases except for faller/non-faller status as a binary, which yielded 75% power.

## Discussion

The key finding of this study is that multiple domains of measurements including gait, neuroimaging, and dual cognitive/motor tasking all provided significant and independent information that explained variability in fall profile variables, including fear of falling and history of falls. The key implication of this finding, if extended to prospective cohorts, is that even if highly sophisticated measurements such as functional MRI of the brain are available, there is still value in a fall risk assessment that is as multi-factorial as possible. The finding emphasizes that falling is influenced by a wide array of factors, including central control of cognitive and motor resources, skeletal muscle function, peripheral nervous system activity, and other contributors not addressed here such as the built environment.

The hypothesis driving our dual-task approach is that in some older adults, performing an additional cognitive task at the same time as standing or walking can reduce the ability to control balance and limb movements to such an extent that the risk of falling is increased. In this way, we were following the lead of a large existing body of literature about dual task performance which posited that interference between disparate task-related cognitive processes (Pashler, 1994), or processing “bottlenecks” (cognitive resources that at any moment can be utilized by one task or the other but not both) (Pashler, 1984) led to decrements in performance on either of the simultaneous tasks. Although we possessed diverse indicators of dual-task decrements in task performance (from task responses, gait performance, and brain functioning), no single type of indicator was predominant in providing the information about fall profile. One possible reason is that the different domains of dual-task performance represent different aspects of dual-task performance, each of which is relevant to falls. Specifically, the cognitive component of the stationary dual task addressed the ability to perform a continuous reactive task that requires constant monitoring, while the cognitive component of the distracted gait task addressed spontaneous language generation. Both types of cognitive performance are relevant to falls and have distinct brain circuitry underpinning them. The imaging variables, meanwhile, address the ability to recruit brain resources from established task-related circuitry to handle the increased demands of the dual task, which could independently contribute to the ability to react to shifting cognitive demands during avoidance of falls. Future work should explore whether there are additional aspects of dual-task performance that could provide even more independent information about fall profile.

The current findings are aligned with, and extend, prior findings on predictors of fall profile variables. The associations between gait variables and fall profile replicate previously published findings in a larger group of individuals from the same LaBrainS cohort (MacAulay et al., 2015), which found that gait step length decreased significantly in the dual task condition compared to the single task condition, and that dual task step length significantly differed between those self-reporting a history of falls vs. those who did not. Dual-task test performance has been shown to be associated with fall risk in many studies with different gait performance measures such as gait variability and gait velocity [for a systematic review, see Muir-Hunter and Wittwer (2016)]. Our study extends these prior results that despite the collection of data from an additional dual task and an fMRI paradigm, these gait task measures remain independently powerful as predictors of fall profile.

Changes in performance and brain functioning between single- and dual-task conditions in a stationary dual task paradigm are well aligned to prior stationary dual-task studies as well. Reaction time in the dual task condition decreased, consistent with multiple dual-task studies (for a meta-analysis, see Verhaeghen et al. (2003). Overall levels of brain activation in task-relevant brain regions reduced in the dual task condition compared to the single task condition as in prior fMRI studies (Bürki et al., 2017), for both the single tapping task and the single AX-CPT task. Our results extend these prior dual-task findings by showing that these dual task decrements in brain functioning and performance are associated with fall profiles. Our research suggests that dual tasking may create a bottleneck in brain resources, as supported by our results that this cohort didn’t seem to prioritize performance of one component of the dual task over the other. Cognitive bottlenecks would imply that the limited amount of attentional resources was spent in trying to do each task “well enough,” resulting in performance deficits in each task instead of a perfect completion of one task. In addition, the ROIs that were a significant predictor of falls have been shown in previous literature to be associated with falls. The right superior parietal cortex is involved in spatial orientation, and has been linked with cognitive-motor dual tasking in previous research (Bürki et al., 2017). The left SMA contributes to movement control, and the DLPMC is associated with motor planning, both of which had a significant correlation with at least one fall variable. A decrease in activation in these areas may contribute to fall risk, since it indicates less attention is put into those specific areas that help with movement.

We did not see a significant correlation between most accuracy and reaction time dual task hits (see Supplementary Table 3). About half of the data seem to cluster along the y-axis, indicating a sacrifice in tapping consistency in order to preserve accuracy. The other half is situated in the upper right quadrant, which represents decrements in both tapping consistency and accuracy. This agrees with findings in a meta-analysis on dual-tasking in older adults (Verhaeghen et al., 2003), which showed that while younger adults will sacrifice either reaction time or accuracy in dual task conditions, older adults will often do the same but can also have decrements in both reaction time and accuracy at the same time. Our data suggests that in the context of our specific stationary dual task, most participants favored accuracy more than reaction time or tapping consistency.

The key strength of this study was its multimodality. Gait characteristics, behavioral measures, and brain activity were all calculated in the same group of individuals to employ each of them as indicators of the fall profile. The main weakness of the study is the self-reported nature of the fall measures, which have known limitations in terms of recall bias. The volunteer participant sample of this study is also not representative (and is not intended to be representative) of the general population, and therefore caution should be applied in extending the results to the general population. The cohort of only 31 participants represents a small sample size but had a relatively high number of fallers so that the study retained a good amount of statistical power. Future work should extend this data *via* longitudinal assessment of the neuroimaging, gait, and behavioral measures as well as assessment within different age ranges and more objective measurement of falls using wrist- or waist-worn devices.

Clinically, a robust, quick, and cheap method to detect falls is preferred. This study does not attempt to replace these methods, but illustrate that there is much information that can be extracted from brain functioning measurements and applied to fall prediction. Since gait, behavioral, and brain imaging measurements provide independent information on fall risk, future work will attempt to provide stratification of fall risk using these multiple measurements domains.

In conclusion, this study found that diverse indicators of gait performance, cognitive and motor performance during a cognitive-motor dual task, and brain functioning during the dual task were all independent correlates of fall profiles in a group of community-dwelling, cognitively-normal older adults.

## Data Availability Statement

The raw data supporting the conclusions of this article will be made available by the authors, without undue reservation.

## Ethics Statement

The studies involving human participants were reviewed and approved by Pennington Biomedical Research Center Institutional Review Board. The patients/participants provided their written informed consent to participate in this study.

## Author Contributions

AV and OC: experimental design. KK, SP, JK, JB, AD, and RB: data collection. KK: data analysis. KK, JK, OC, AV, and JB: data interpretation. KK and OC: manuscript drafting. All authors contributed to editing the manuscript.

## Conflict of Interest

The authors declare that the research was conducted in the absence of any commercial or financial relationships that could be construed as a potential conflict of interest.
