# Supplementary material for: Neuroimaging, Behavioral, and Gait Correlates of Fall Profile in Older Adults
Source: Front Aging Neurosci. 2021 Feb 18;13:630049. doi: 10.3389/fnagi.2021.630049 (PMC7935539; doi:10.3389/fnagi.2021.630049)
Supplement: Supplementary file 1 [file Data_Sheet_1.PDF]

AX-CPT ROIs	MNI X	Y	Z	Reference
L superior parietal cortex	-50	-70	50	Lesh, Tyler A., et al.
R DLPFC	52	12	36	Lesh, Tyler A., et al.
R inferior parietal cortex	50	-44	50	Lesh, Tyler A., et al.
L DLPFC	-48	32	24	Lesh, Tyler A., et al.
L inferior parietal cortex	-44	-52	42	Lesh, Tyler A., et al.
R superior parietal cortex	34	-68	44	Lesh, Tyler A., et al.
R DLPFC	56	28	28	Lesh, Tyler A., et al.
R ACC SMA	2	22	48	Lesh, Tyler A., et al.
R middle frontal gyrus	48	12	26	Lopez-Garcia, Pilar, et al.
R insula	32	22	0	Lopez-Garcia, Pilar, et al.
L SMA	-16	-2	64	Lopez-Garcia, Pilar, et al.
R fusiform gyrus	44	-50	-18	Lopez-Garcia, Pilar, et al.
R inferior parietal cortex	30	-68	44	Lopez-Garcia, Pilar, et al.
L fusiform gyrus	-40	-54	-14	Lopez-Garcia, Pilar, et al.
L inferior parietal gyrus	-26	-70	40	Lopez-Garcia, Pilar, et al.
L middle frontal gyrus	-46	4	26	Lopez-Garcia, Pilar, et al.

Motor cortex ROIs	X	Y	Z	Reference
L sensorimotor cortex	-38	-29	53	Witt, Suzanna, et al.
R sensorimotor cortex	-36	-25	57	Witt, Suzanna, et al.
Supplementary motor area	-4	-11	56	Witt, Suzanna, et al.
L ventral premotor cortex	-55	-4	35	Witt, Suzanna, et al.
L inferior parietal cortex	-51	-28	20	Witt, Suzanna, et al.
R inferior parietal cortex	40	-46	45	Witt, Suzanna, et al.
L basal ganglia	-22	-8	4	Witt, Suzanna, et al.
R basal ganglia	22	-11	6	Witt, Suzanna, et al.
L anterior cerebellum	-22	-55	21	Witt, Suzanna, et al.
R anterior cerebellum	16	-50	-27	Witt, Suzanna, et al.
R dorsal premotor cortex	47	-2	54	Witt, Suzanna, et al.
R dorsal premotor cortex2	38	-13	58	Witt, Suzanna, et al.
R dorsolateral prefrontal cortex	42	33	37	Witt, Suzanna, et al.
R dorsolateral prefrontal cortex2	32	29	36	Witt, Suzanna, et al.
R inferior parietal lobe	46	-48	45	Witt, Suzanna, et al.
L ventral premotor cortex2	-53	-3	11	Witt, Suzanna, et al.
R posterior cerebellum	10	-66	-42	Witt, Suzanna, et al.
L dorsal premotor cortex	-16	-23	51	Witt, Suzanna, et al.
R dorsal premotor cortex3	34	-11	56	Witt, Suzanna, et al.
R dorsal premotor cortex4	46	-2	55	Witt, Suzanna, et al.
L ventral premotor cortex3	-57	-6	37	Witt, Suzanna, et al.
L ventral premotor cortex4	-55	2	11	Witt, Suzanna, et al.

L ventral premotor cortex5	-51	-2	37	Witt, Suzanna, et al.
R inferior parietal cortex2	46	-48	45	Witt, Suzanna, et al.
R inferior parietal cortex3	38	-43	46	Witt, Suzanna, et al.
L posterior parietal cortex	-26	-66	49	Witt, Suzanna, et al.
R posterior parietal cortex	16	-76	42	Witt, Suzanna, et al.

Supplementary Table 1. List of each ROI used in fMRI analysis.

	Single	St. error	Dual	St. error	% diff	p-value
Accuracy (all trials)	94.6	1.4	82.6	3.6	-12.7	< 0.001
AX	94.0	1.8	80.7	1.4	-14.1	< 0.001
BX	96.0	1.9	84.7	4.0	-11.8	0.01
AY	95.2	1.6	87.1	1.8	-8.5	0.01
BY	99.2	0.8	94.4	1.9	-4.9	0.11
Reaction time (all trials)	0.74	0.04	0.80	0.04	9.0	0.04
AX	0.74	0.05	0.80	0.02	7.5	0.13
BX	0.80	0.04	0.86	0.01	7.3	0.19
AY	0.74	0.04	0.81	0.05	10.2	0.03
BY	0.59	0.02	0.78	0.04	31.8	< 0.001

Supplementary Table 2. Accuracy and reaction time changes for each trial type for single AX-CPT task and dual task.

	Accuracy decrease: (D-S)	% difference in RT: (D-S)/S * 100
% Difference in tapping STD (D-S)/S * 100	-0.07 (0.72)	0.27 (0.13)
Change in tapping cadence, D-S	-0.10 (0.59)	0.41 (0.02*)
% Difference in tapping cadence (D-S)/S * 100	-0.12 (0.51)	0.38 (0.04*)
Change in STD, D-S	0.04 (0.83)	0.25 (0.17)

Supplementary Table 3. Correlation coefficients between AX-CPT dual task hit and tapping dual task hit, with p-values in parenthesis. Significant ($p < 0.05$) values are marked with *, STD = standard deviation. D represents dual task, and S represents single task.

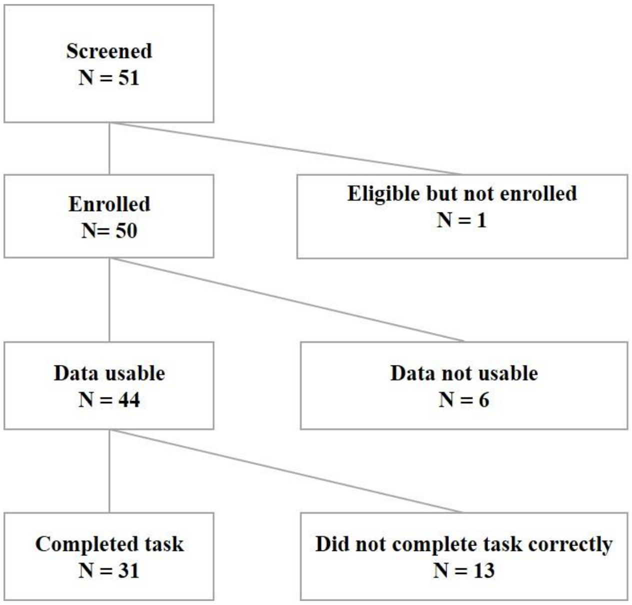

Eligible but not enrolled	Data not usable	Did not complete task correctly	Reason
1			Dropped from study
	4		Technical error; no data recorded
	2		Excessive head motion (< 30%)
		5	Fell asleep
		4	Did not tap during dual task
		4	Did not respond to enough trials

Supplementary Figure 1. Consort diagram for LABS study participants.

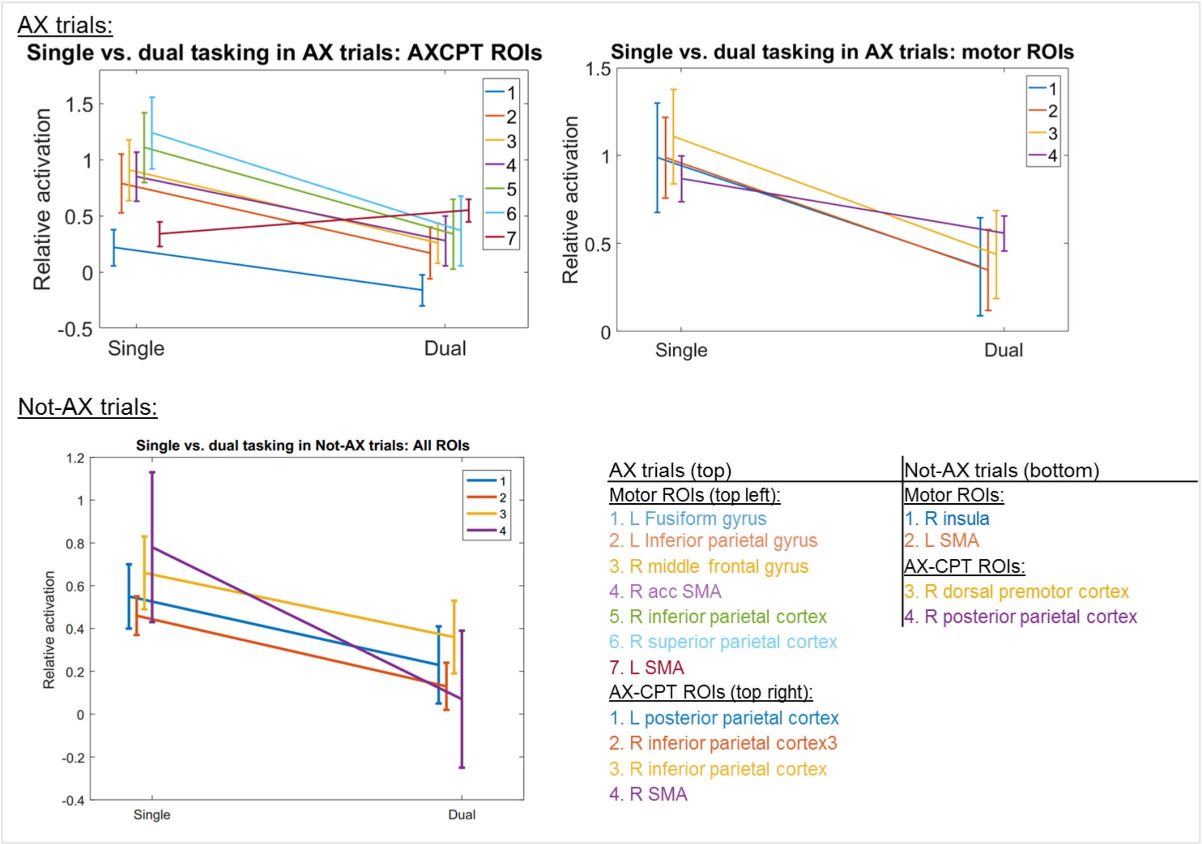

Supplementary Figure 2. Imaging data for each contrast of AX trials and not-AX trials. For the AX trials, ROIs are split into motor and AX-CPT ROIs. For the not-AX trials, (AX) indicates an

ROI that was present in the set of AX-CPT ROIs, and (M) indicates one in the set of motor cortex ROIs.
